# Complexity and Variation in Infectious Disease Birth Cohorts: Findings from HIV+ Medicare and Medicaid Beneficiaries, 1999–2020

**DOI:** 10.3390/e26110970

**Published:** 2024-11-12

**Authors:** Nick Williams

**Affiliations:** Lister Hill National Center for Biomedical Communications, National Library of Medicine, National Institutes of Health, 8600 Rockville Pike, Bethesda, MD 20894, USA; nick.williams@nih.gov

**Keywords:** HIV, human immunodeficiency virus, information theory, correlation and variance, complexity, medicare, medicaid, child health insurance program, age, period, cohort, regression, survival analysis, clinical demography

## Abstract

The impact of uncertainty in information systems is difficult to assess, especially when drawing conclusions from human observation records. In this study, we investigate survival variation in a population experiencing infectious disease as a proxy to investigate uncertainty problems. Using Centers for Medicare and Medicaid Services claims, we discovered 1,543,041 HIV+ persons, 363,425 of whom were observed dying from all-cause mortality. Once aggregated by HIV status, year of birth and year of death, Age-Period-Cohort disambiguation and regression models were constructed to produce explanations of variance in survival. We used Age-Period-Cohort as an alternative method to work around under-observed features of uncertainty like infection transmission, receiver host dynamics or comorbidity noise impacting survival variation. We detected ages that have a consistent, disproportionate share of deaths independent of study year or year of birth. Variation in seasonality of mortality appeared stable in regression models; in turn, HIV cases in the United States do not have a survival gain when uncertainty is uncontrolled for. Given the information complexity issues under observed exposure and transmission, studies of infectious diseases should either include robust decedent cases, observe transmission physics or avoid drawing conclusions about survival from human observation records.

## 1. Introduction

This study attempts to detect anomalies using information theory concepts in at-scale life course data for people living with HIV in the United States. People living with HIV are very different from each other for multiple under-observed and non-trivial reasons that likely impact any attempt at evaluating affected populations. Population sample studies often attempt to control for under-observed features, but their scale (small) is often not representative of the wider population over time. In turn, answering population level questions, such as, “is HIV epidemic or endemic in the United States”, or “is survival improving”, becomes fundamentally difficult to answer due to under-observed and out-of-scale local features.

Theoretically, anomalies should exist in any series of information if underlying variance is present. Said variance could be caused by real world improvements to treatment efficacy, and access or a worsening of ground conditions from viral resistance to treatment or other effects. Age-Period-Cohort (APC) is a logical choice to explore anomalies in temporal reference network data due to the availability of period and birth cohort features in at-scale clinical data. There is precedent for APC’s usage to explore Temporal Reference Network problems among the ‘global burden of disease studies’ (GBDS) that compare clinical conditions within countries across nations and over time [1,2,3,4]. The data used here is more specific than GBDS because it is sourced from identifiable patient-encounter-level records, whereas GBDS uses publicly available aggregates.

Using APC disambiguation, we observe the variance, if any, between HIV+ cases across birth cohorts and over time as they age. Detected anomalies should implicate changes in how people are living and dying over time. The absence of variance in a temporal reference network likely indicates that a continuous era is present on the ground despite political or small-scale observations. Emergent survival time stability is one such concern, as are the case stability (endemic) or case variation (epidemic) conditions in this study. The use case presented here is not comparative, but anomaly detection, where the absence of an anomaly indicates a stable era where cases are aging and dying in similar ways over time. Such mortality stability, if detected, would preclude treatment improvement macro effects, and case stability would indicate stable endemic effects. To consider mortality stability, decedents observed dying over the study period are handled separately from survivors to better understand observed survival time.

Emergent survival time and case variation in an infectious disease (ID) cohort is a classical information theory signal problem but is seldom remembered to be one [5]. Information theory signal problems are typically problems of signal interpretation assessed or resolved through observations of the physics of transmission, noise and receiver states. Information theory for infectious diseases could be thought of as a discrete infectious agent (signal) provoking a common physiology–pathology (physics) via infection (transmission) to a person with preexisting and forthcoming pathologies (noise) observed at different points in time (era) across very different hosts (receivers). Evaluating simple questions with complex materials such as, ‘are HIV+ patients living longer,’ or ‘is HIV epidemic or endemic,’ requires engagement with signal complexity, implicit human observation subunits and inherent materials being observed (human observation records).

Human observation records as a material site of complexity can inform solutions to signal problems by pre-classifying information (even erroneously) and then observing the physics of an information theory of Transmission, Receiver or Noise (TRN). Such a theory can be proposed and evaluated. This proposal and evaluation are constructive and will likely generate an informed classification scheme to produce a natural history of information, or a true signal interpretation of a given ID-TRN relationship. Measuring individuals over time within populations is one way of drawing conclusions about TRN; but, evaluating the quality of those conclusions should still be pursued to improve a given model.

Because the underlying unit of ID analysis is a person who becomes infected, transmits and dies, mortality, aging and observation period are key dimensions in any evaluation of demic or survival states. But TRN physics are seldom observed or reported in ID or HIV studies. As a candidate physics system to manage TRN, APC may be of some use [6]. APC is the disaggregation of an observed effect by the age (at observation), (time) period observed and (birth) cohort the observation was sampled from or is attributable to. The core concern of APC is that different time periods, ages and different birth cohorts will have different effects on observed physics because age confers vulnerability, period confers era and birth confers origination effects. APC confounding occurs when conclusions drawn about units are false because of an under appreciation of the subunit age, time period and origination effects [4,7,8]. Whenever similar phenomena are being observed over time, APC can assist in the correct interpretation of known, and under-observed, TRNs.

Several authors have, over the last 20 years, used non-systematic, convenience samples with unresolved TRN physics to conclude that HIV patients have improved survival rates despite declines in life expectancy nationally in the United States [9,10,11]. How detectable these effects are in an at-scale, human observation record system, like the Centers for Medicare and Medicaid Services (CMS), remains under-described.

With no clear bright line between epidemic or endemic HIV conditions, the United States warrants specific engagement with birth cohort risks [12,13]. It may be that prevention tools are unfit for use if HIV in the 21st century in the United States is endemic rather than a series of geographic, resource and racial epidemics, as in the 20th century [14,15,16,17]. Without concrete and universal surveillance capture of date of infection (not diagnosis), alternative measures should be considered to benchmark outcomes. Year of birth, or as it is known in epidemiology circles, ‘birth cohort’ may be one such measure. Year of birth is a recoverable data point (in most settings) which likely has high recall and verification potential.

There is also a curious anthropological question inherent in HIV-TRN problems; specifically, as HIV is a blood-borne pathogen and sexually transmissible disease. Maternity confusion (confusion regarding to whom or when one was born) is rare in human societies relative to paternity confusion (regarding by whom or when one was inseminated). This distinction between paternity confusion (common) and maternity confusion (rare) has implications for the kinds of information available in human observation records. While paternity confusion (frequent, lack of witnesses, unattended) is common and maternity confusion is not (once per lifetime, wealth of witnesses, unattended birth is rare) the availability of maternity data to draw inferences from paternity events could be a wider opportunity to interrogate TRN physics by considering their maternal (lower entropy), rather than paternal (higher entropy), observations. Gorban, A. N.; Tyukina, T. A.; Pokidysheva, L. I.; and Smirnova, E. V. argue that when interpreting complex information, especially adaptation cost, variance and correlation is required; yet, in the case of HIV-TRN problems or wider ID-TRN problems, when the subject was born may create an artificial, modulating measure against which candidate’s hidden layer paternal signals could be plotted and interrogated [18].

Here, we consider APC effects on observations of HIV+ cases and deaths among human observation records from Medicare and Medicaid, 1999–2020. Medicare is a SafetyNet insurance program that provides payment for clinical care for adults over the age of 65 and permanently disabled individuals. Medicaid is a collection of federal/state matching dollar insurance programs administered by states with highly varied eligibility criteria. Medicaid claims include Child Health Insurance Program (CHIP) records as well. CMS administers Medicare and Medicaid payments; CMS provides payment for 39% of health expenditure in the United States and provided varying levels of coverage to 160 million people in 2022 [19]. Medicare and Medicaid are the nation’s largest payers for HIV care [20,21].

This study finds that as they age, millennial HIV+ cases known to CMS appear on-track to have the same (bad) outcomes (HIV infection) as their baby boomer forbears. In this study, as younger birth cohorts accrue time, they are expected to present with similar HIV case volumes and mortality volumes as older birth cohorts and they do present so. Consequently, the epidemic era in the United States could be over. It would be more accurate to say HIV is endemic to the United States, or regularly occurring due to common local conditions rather than rare foreign, vector-specific ones, within birth cohorts with transmission and age-specific risk profiles. Further, this study does not detect generalizable survival gains for the national population except in very rare, specific kinds of patients (HIV+ children, very old adults). This study also detects co-morbid mortality impacts, likely due to the COVID-19 pandemic and influenza mortality. Living longer is perhaps an oxymoron, as an individual can only die once, in one given moment, the complexity/adaptation cost of which is immutable and non-transferable. It could be that the use value of cox regression is political, rather than clinical.

## 2. Materials and Methods

Study data were sourced from the Chronic Conditions Warehouse, Virtual Research Data Center; a 100% sample of Medicare and Medicaid research-identifiable file records for the study years 1999–2020 were considered. Records were eligible for the study if they were attributable to an individual. Do note that Medicare records detail many individual beneficiaries with life course durations which exceed popular expectations (observed over age 110), and Medicaid attributes maternal care to pre-birth cases who go on to be born as CHIP beneficiaries (observed at years old at observation 0 or −1). Being observed at birth year zero is practical in this study, as is being observed at age 110+. Individuals with ‘implausible’ birthdates were considered, but only individuals who could be assigned a ‘Years Old At Observation (year)’ or YOAO within study years (1999–2020) between 0 and 100 years old were included in this study. Consequently, individuals observed more than one year before their date of birth and individuals observed more than 100 years after their birthdates had their over- and under-reaching observation years excluded. Cases with over-reaching and under-reaching observation years were considered as such, and their study-qualifying years included.

The study case index was created out of individuals described in Medicare or Medicaid records who were either ‘ever HIV+’ on a Medicare or Medicaid claim from 1999 to 2020 or ‘never HIV+’ in a study period claim. Qualifying as ‘ever-positive’ was assigned through ICD9-CM code 042 or ICD10-CM codes Z21 or B20. No ‘ground truth’ was sought to verify HIV status for beneficiaries. This case definition for HIV+ is fairly broad; and traditionally studies of the HIV+ CMS population use more restrictive case definitions to ensure the HIV cases captured are fit for use in complex research models [22,23,24].

Because APC analysis only requires a date of birth, a year observed, date of death (if dead) and a status (to disambiguate cases and controls), this general HIV case definition is likely sufficient. More complex study models may benefit from a more restrictive definition. It is likely that individuals who are considered HIV− in this study could survive to contract HIV and have their HIV status become ‘known’ to Medicare or Medicaid, or are infected with HIV and simply never sought HIV care or are not (yet) known to Medicare or Medicaid. In turn, conclusions about the HIV− cohort should also be made with care. Findings from the HIV− cohort, specifically their APC effects, are not presented here.

The case index contained date of birth, service observation year, subject ID, date of death, date of enrollment (for Medicare), years observed (submitting claims) and HIV+ ever status. One index was created for each program, Medicare and Medicaid. A third index was also created out of distinct case-age-observation year triplets to integrate multiple payor programs as a distinct population. In the integrated index (referred to as CMS below), cases could have one observation year per study year, even if they were dual enrolled in the observation year (in both Medicare and Medicaid administered programs). The study dataset aggregated the case indexes by YOAO and then counted cases and decedent cases observed within the observation year by HIV status and program (Medicare, Medicaid or CMS). Small cells where the number of case observation years was less than 11 were redacted (set to 0) to remain in compliance with CMS privacy standards.

## 3. Results

The study analyses are meant to detect anomalies within age, period and birth cohort dynamics when evaluating APC as a proxy for TRN physics among HIV+ cases and HIV+ decedents in the United States who are known to CMS. Of particular interest are anomalies that inform interpretation of survival time and demic status. Four analyses are presented here. First, we review the underlying data for context and to familiarize readers with CMS data. Second, we consider case inflow, and overflow dynamics, using two factor APC plots to relay the stability of human observation records. Third, we observe cases and decedents directly using two factor APC plots. Lastly, we consider a statistical model evaluating stability among mortality cases.

### 3.1. CMS Data Considered in This Study

**Figure 1:**
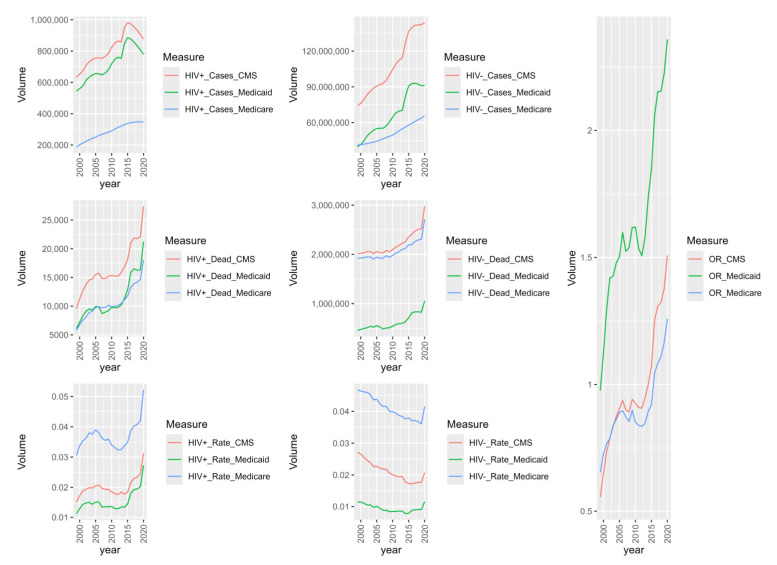
CMS case volumes over time by death with mortality rates and mortality relative rate (OR) by HIV status.

The study analysis first considers summary counts of case attribution by diagnostic code over study observation years (Table 1), followed by cases, deaths and observation years in total by program (Table 2) and by CMS group over time (Table 3). Figure 1 plots each case index over time.

In Table 1, individuals can be counted once per diagnostic code per year. Diagnostic code utilization appears stable, except in the ICD-9CM to ICD-10CM transition period (2015). Some providers still bill ICD9-CM HIV code 042 well into the 2020 observation year. HIV cases were qualified as HIV+ if they ever had qualifying HIV codes on any program. Do note that Z21 is ‘a-symptomatic’ HIV, and B20 is meant for AIDS diagnosis in the ICD10-CM era. Aspects denominated by ‘0’ in Table 1 are not truly zero but contain fewer than 11 cases and were redacted to comply with dataset specific privacy policies.

Table 2 shows an almost 100% difference in the size of programs, with Medicaid at 207 million distinct individuals considered and Medicare at 109 million individuals. Medicaid has almost three times the distinct HIV+ volume of Medicare (.580 hundred thousand vs. 1.44 hundred thousand). Nearly all (save 96,751, or 16.66%) Medicare HIV+ cases will be observed on Medicaid at some point over the study period. Four in ten HIV− Medicare cases died over the observation period, with a similar proportion for the Medicare HIV+ group. The HIV+ group within Medicaid are nearly three times more likely to be observed dying than their HIV− counterparts. HIV+ Medicaid cases have longer observation periods than their HIV− counterparts; parity is observed for Medicare. Observation years among decedent cases also have parity for Medicare and are slightly longer for Medicaid. Combining Medicare and Medicaid records provides 1.5 million HIV+ life courses and 0.363 million HIV+ deaths for analysis.

Mortality rates peak twice in Table 3, first in 2006 and again in 2020. The elevated 2020 mortality may be due to COVID-19 pandemic impacting cases. In turn, there could be a ‘hidden peak’ in 2019 where COVID-19 mortality is not yet available as a mortality drain for the HIV+ cohort. HIV+ cases observed peaks in 2015, and a deceleration is observed through study year 2020 in case observation.

**Figure 2:**
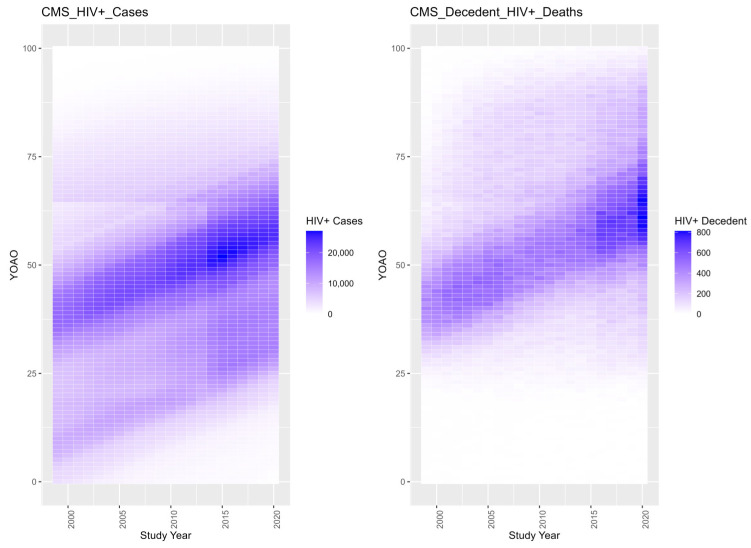
Tile plot of CMS HIV+ cases and deaths by number of years old at observation (YOAO).

Figure 2 plots HIV+ and HIV− cases, decedent mortality rates and the mortality relative rate (OR) over study time. HIV cases peak in Medicaid in 2014 and decrease towards the end of the study period, perhaps due to Medicaid expansion. HIV+ mortality has three peaks when programs are disambiguated, in 2006, 2016, and in 2020 for HIV+ cases. HIV− mortality appears stable over time until the 2020 study year. HIV− mortality rates decrease over the study period, but spike in 2020 while HIV+ mortality rates rise and fall for the Medicare population across the study period. All HIV+ mortality ORs reach HIV+ overburdened (greater than 1) by 2016, though Medicaid HIV OR is always above parity. HIV− cases have increasing case observation, though Medicaid case observation plateaus, possibly due to Medicaid expansion during the study period. Decedent cases spike in 2020 for all populations, perhaps due to COVID-19.

### 3.2. Observation Stability Dynamics

Next, the study considers more complex data physics with tile plots of HIV+ case and HIV+ decedent capture (Figure 2). Figure 2 indicates that multiple populations of HIV+ cases are being observed with different life courses and end points. Note bimodal case observation before and after age 65. A bimodal distribution for mortality shows an early and a late study death group. Observation years for HIV+ cases and deaths show a distribution where cases are observed until age 65, suggesting that the CMS population enrolls and dies out prior to age 65 and then is ‘back-filled’ with 65-year-olds enrolling in Medicare and older adults contracting HIV late in life. Deaths are observed across ages and the under-25 cases experience mortality. It becomes more difficult over time to observe pediatric HIV+ cases. Mortality is elevated in 2020. Medicaid expansion in 2014 is likely the cause of the case volume increase observed in 2014.

**Figure 3:**
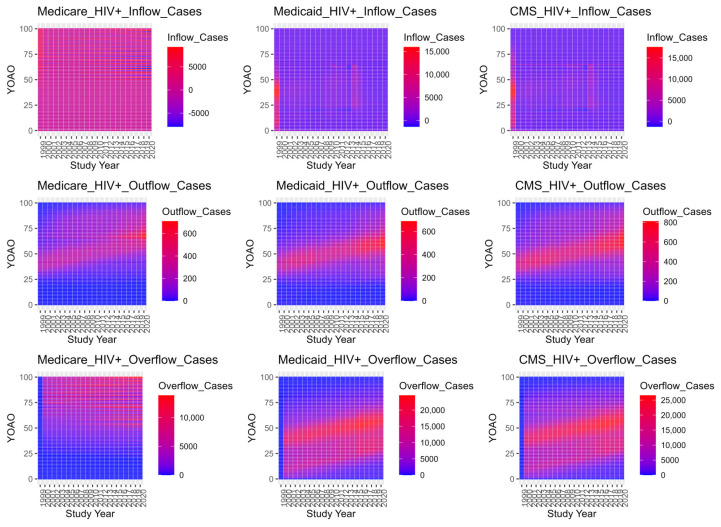
Inflow candidates, outflow (death) and overflow candidate case volumes by program and across study years by number of years old at observation (YOAO).

Inflow, outflow (death) and overflow candidate dynamics are also presented as tile plots. Figure 3 details the outflow, overflow and inflow candidacy dynamics by their YOAO and study year. Outflow cases are decedent cases who are ineligible for observation in the following study year and YOAO step. Overflow candidates are cases involving an individual who did not die in the prior YOAO tile over the study time and is eligible for the following YOAO and study year. Inflow candidates are cases in which the individual was observed and could not have overflowed from prior cells: most likely new or return enrollees. Inflow is the current YOAO and study year subtracted from the overflow, to produce candidate inflow case volumes who could not be overflow cases. HIV+ inflow candidates can have negative number ranges because mortality is highly concentrated in YOAO cells, and disenrollment mechanics which confound observation year capture are detected.

Outflows in Medicare are bimodal with a low band for 64-year-olds, suggesting cases where individuals survive to 64 and are backfilled by new enrollees at 65 YOAO. Medicaid outflows occur in two distinct groups as well, with a low decedent period from 2009 to 2014. CMS outflows lose the Medicare backfill effect, suggesting that some HIV+ Medicaid cases join Medicare at 65 and conceal prior Medicare deaths at 60–64. Medicare overflow cases are most common after age 50, while Medicaid overflow is trimodal. CMS overflow retains features of Medicare and Medicaid overflow, suggesting underlying disease dynamics are not resolved with data integration. Medicaid inflow cases show negative values at the age of 19–20, indicating that CHIP disenrollment impacts HIV+ ever case observation years, perhaps prior to infection, though perhaps not. Medicaid expansion may be responsible for the dense bands from 2014 onward in Medicaid HIV+ inflows. CMS HIV+ inflow shows both the high band across study years at 65 years old (perhaps traditional Medicare enrollment), and low band at 19 years (CHIP exodus).

### 3.3. Two by Two Plots of Interactions Between Age, Period and Cohort Effects

Next, the study considers the relationships between APC effects themselves. Plots of two Age-Period-Cohort indices are provided for inflow, overflow, outflow and case volumes. As above in Figure 3, cases are the baseline measurement for cases observed in a given year, age, or birth cohort. Outflow is the explicit mortality among CMS HIV+ cases. Overflow candidacy was calculated as year prior cases minus the prior year’s mortality. Inflow candidacy refers to cases that cannot be overflow cases, or next year’s cases minus overflow. Inflow can be a negative number.

#### 3.3.1. Inflow Candidates or Cases Entering the Study

**Figure 4:**
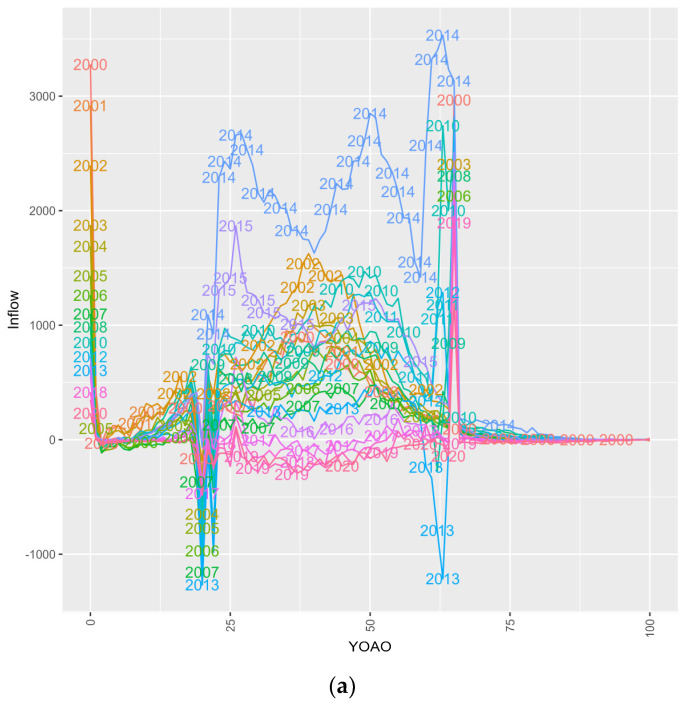

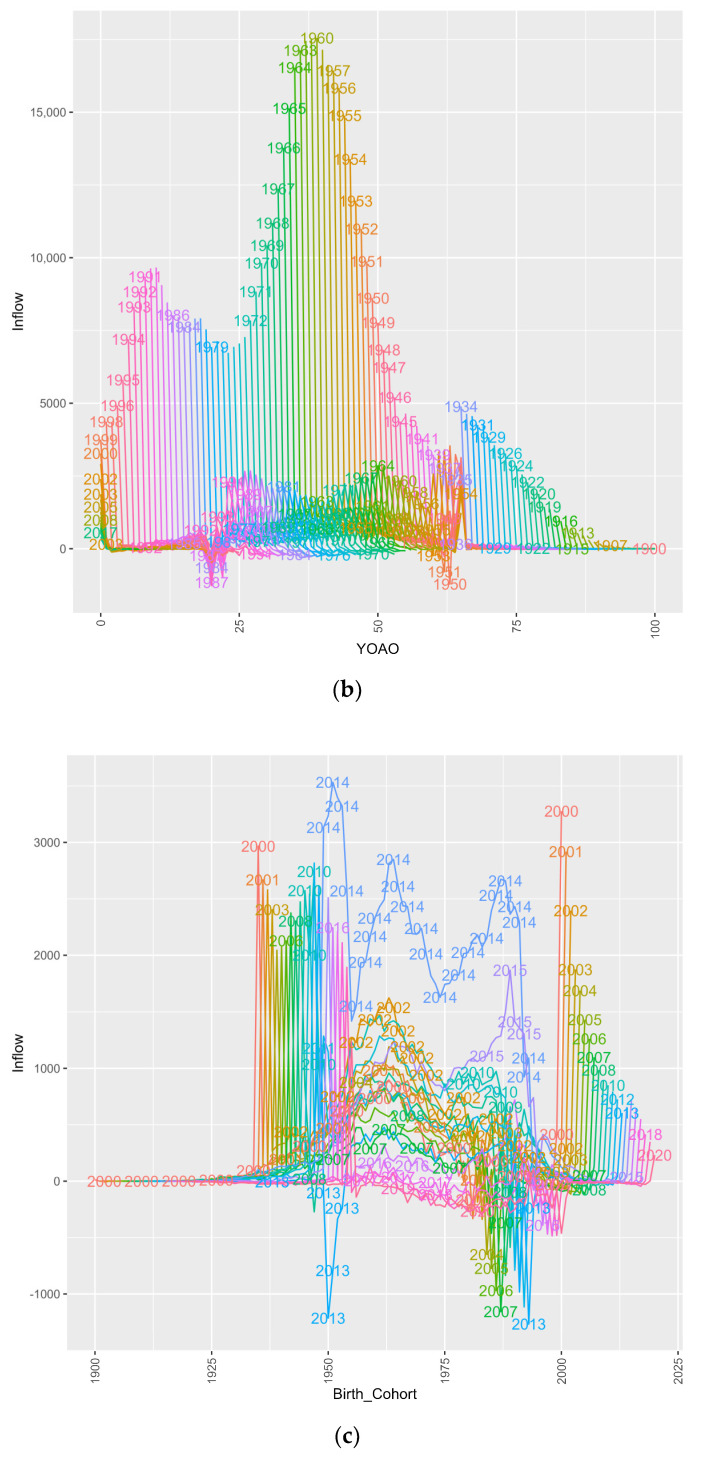

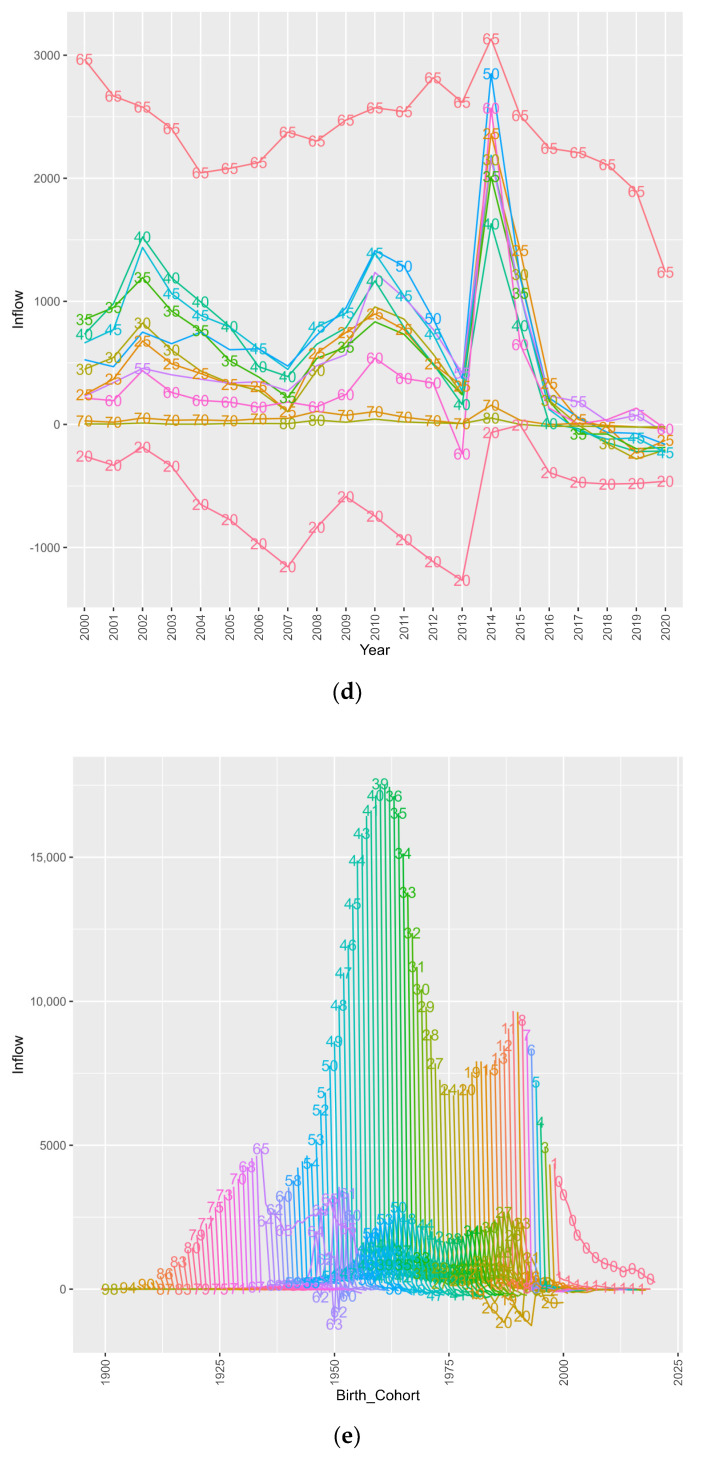

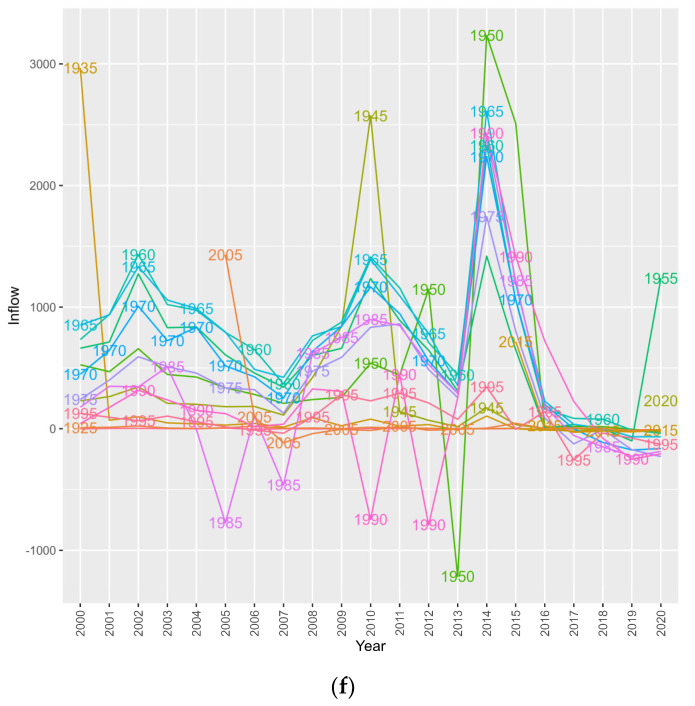
(**a**) Inflow among CMS HIV+ cases, YOAO in period. (**b**) Inflow among CMS HIV+ cases, YOAO in cohort. (**c**) Inflow among CMS HIV+ cases, cohort per period. (**d**) Inflow among CMS HIV+ cases, period in YOAO. (**e**) Inflow among CMS HIV+ cases, cohort in YOAO. (**f**) Inflow among CMS HIV+ cases, period in cohort.

Figure 4 subplots inflow, or HIV+ CMS cases entering this study by two factor APC plots, considering two age, period or cohort features at a time for a total of six subplots. Key points are summarized below on a subplot-by-subplot basis. 

Subplot 4a shows the inflow in the study period within the age for HIV+ CMS cases. Spikes are observed for ages 19 and 65, reflecting Medicare enrollment and CHIP disenrollment dynamics. In early study years, inflow is highest at age 40, and in later study years inflow has peaks after age 20 and at age 50. The 2014 anomaly may be caused by Medicaid expansion. Most inflow candidates are 25–64 years old.

Subplot 4b shows the inflow for birth cohort relative to age. Cases that are born in CHIP eligible birth cohorts have CHIP disenrollment effects, and birth cohorts that are eligible to be age 65 in this study have Medicare enrollment effects. Younger birth cohorts are observed at younger ages, while middle and late birth cohorts follow a trimodal distribution (peaks at age 10, 35 and 65). There is an additional subset, between 25 and 64, under 5000 cases. This lower group has different inflow dynamics relative to the primary, trimodal population. They could be non-public or Ryan White AIDS cases moving to Medicare via Social Security Disability Insurance or may have other explanations.

Subplot 4c shows the inflow of study periods by birth cohort. Older birth cohorts were present in a distinct region on the left side, from 1925 to 1950. A middle group ranges from the years 1950 to 2000 and a final group is observed over the study years themselves. This first group are older adults who most likely contracted HIV very late in life. The middle group are observed as adults. This group has such acute mortality (outflow) that the inflow volume cannot be explained from overflowing cases, hence the negative adult region values. The birth cohorts that are included in study years are indicated as pediatric HIV cases, as they are too young to have contracted HIV before the study years. The comparative variance in the adult group is likely due to the different ages at infection and diversity of kinds of HIV infected cases (i.e., otherwise healthy adults, AIDS cases, Intravenous Drug Users, Hepatitis C+ cases).

Subplot 4d shows the inflow of select ages within study periods. Medicaid expansion in 2014 increases case observation year volume. There is a dual peak space between 2002 and 2011 which could have a third peak that is possibly affected by the addition of expanded Medicaid cases. Note that age 65 and age 20 appear linear, indicating a regular inflow of cases at 65 and a negative inflow of CHIP cases.

Subplot 4e shows the inflow of ages within birth cohorts. The trimodal distribution conceals a subgroup of cases from birth cohort 1945 through 1995. Older adults, adult and pediatric cases are clearly differentiated.

Subplot 4f shows the select inflow of birth cohorts within study periods. The impact of Medicaid expansion in 2014 is observed. Birth cohort ‘high points’ co-occur when individuals would be 65 years old, indicating Medicare inflow. Pediatric birth cohorts leave the model during expected CHIP exodus.

#### 3.3.2. Overflow Candidates or Cases Moving Through the Study

**Figure 5:**
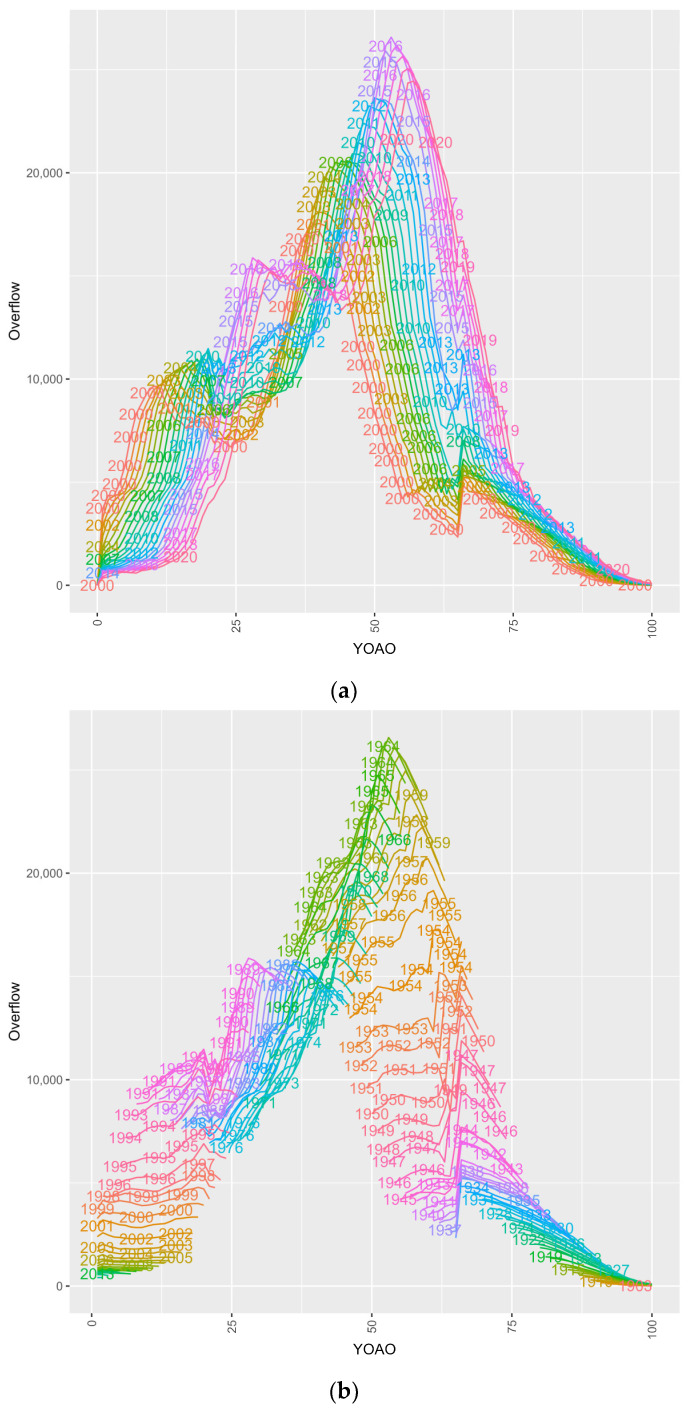

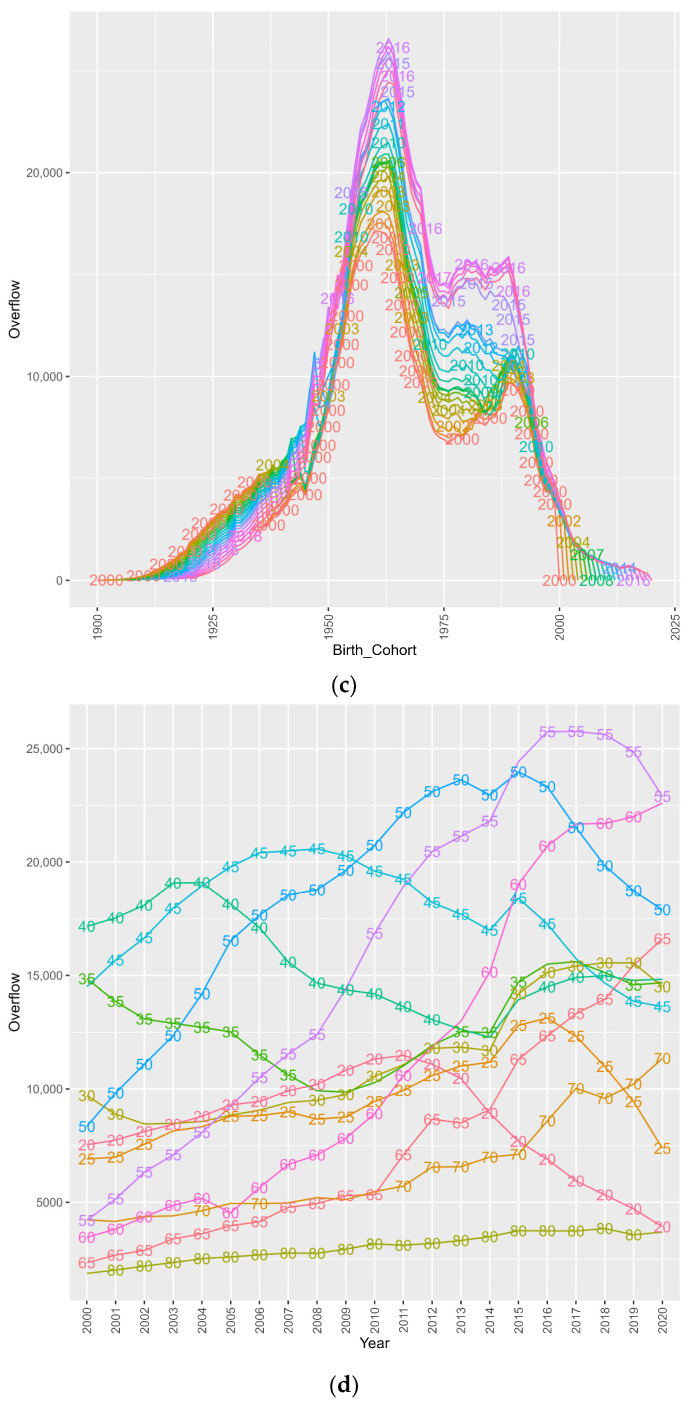

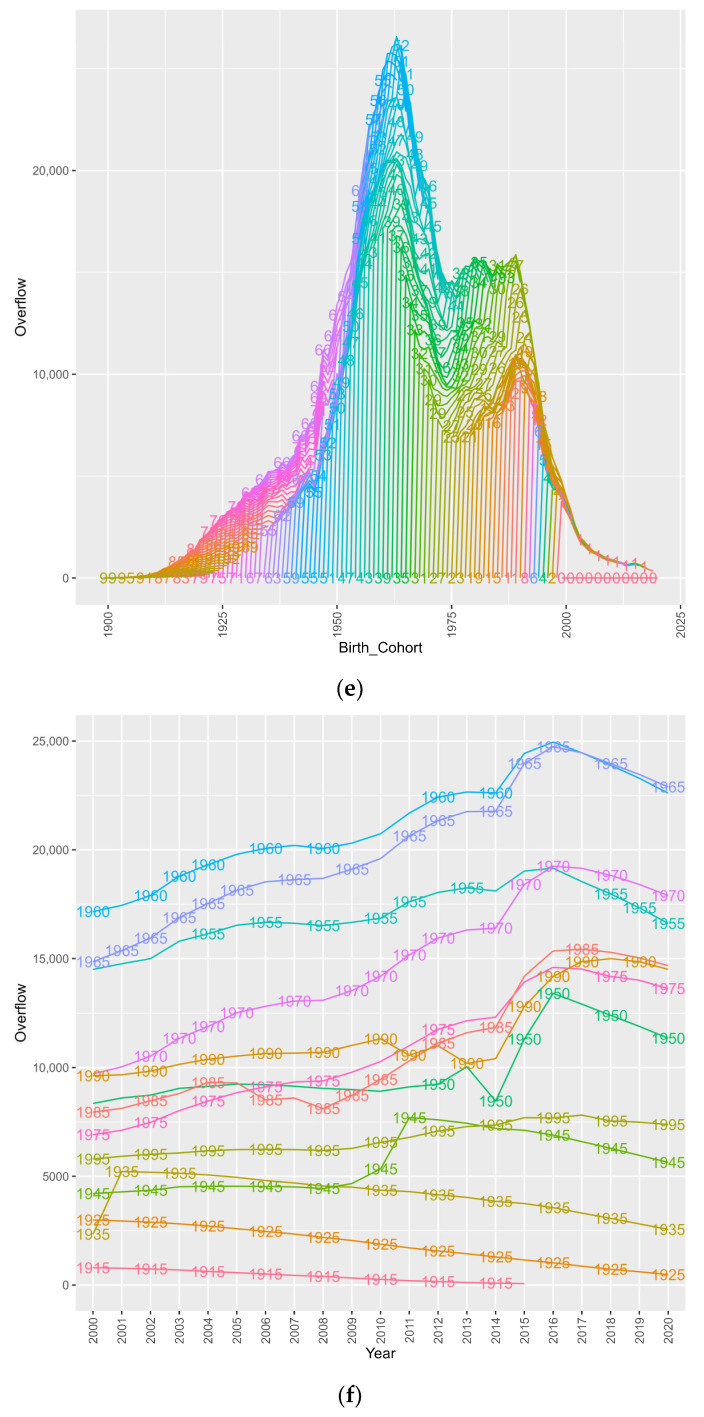
(**a**) Overflow in CMS HIV+ cases, YOAO in period. (**b**) Overflow in CMS HIV+ cases, YOAO in cohort. (**c**) Overflow in CMS HIV+ cases, cohort in period. (**d**) Overflow in CMS HIV+ cases, period in YOAO. (**e**) Overflow in CMS HIV+ cases, cohort in YOAO. (**f**) Overflow in CMS HIV+ cases, period in cohort.

Figure 5 subplots overflow, or HIV+ CMS cases observed year on year in this study by two factor APC plots, considering two age, period or cohort features at a time for a total of six subplots. Key points are summarized below on a subplot-by-subplot basis.

Subplot 5a shows the overflow of study periods within age ranges. Early and middle study years follow a three-peak distribution between 0 and 25, 25 and 50 and at 65. Later study years show a movement of the first and second peaks from 0–25 to 25+ and 25–50 to 50+. This may indicate cases breaking through previous survival ceilings or individuals becoming infected and being observed later in life. The 25+ anomaly is likely due to Medicaid expansion, and prior years are likely missing from these epidemic cases as they were not observed in CMS but likely existed in the real world.

Subplot 5b shows the overflow of birth cohorts by age. Cases at age 50 have the greatest overflow (aging through) followed by cases aged 25. The younger birth cohorts did not have as much ‘time’ to be observed as adults as of this writing. The decrease in observing overflow over birth cohort time may indicate that cases are reaching their survival ceilings and leaving the model (outflow) when their birth cohort lines peak (start dropping). Birth cohorts also see overflow peaks once they qualify for Medicare at 65.

Subplot 5c shows the overflow of study periods within birth cohorts. Distributions are consistent across study years with peaks in the 1960 and 1990 birth cohorts for overflow case observation. Note that later study years contributed more cases than expected from cohorts 1975–2000. This may indicate another wave of transmission which is impacting younger people.

Subplot 5d shows selected ages within study period for overflow cases. The overflow in 40- and 35-year-olds drops over the study period and increases in 2014. In 55–70-year-olds, there is increased overflow over the study period. Further, 20- and 25-year-olds have decreased overflow, while 30-year-olds have a consistent increase.

Subplot 5e shows ages within birth cohorts. Age within birth cohorts appears normal given the progression of study time. Birth cohorts from 1975 to 1990 show increases, perhaps suggesting that the baby boomer epidemic and millennial HIV have different observational mechanisms.

Subplot 5f shows select patterns in overflow by birth cohort within study periods, with normal distributions observed throughout. Note the decreases in very old birth cohorts (1915–1935).

#### 3.3.3. Outflow Candidates or Cases Leaving the Study Due to Death

**Figure 6:**
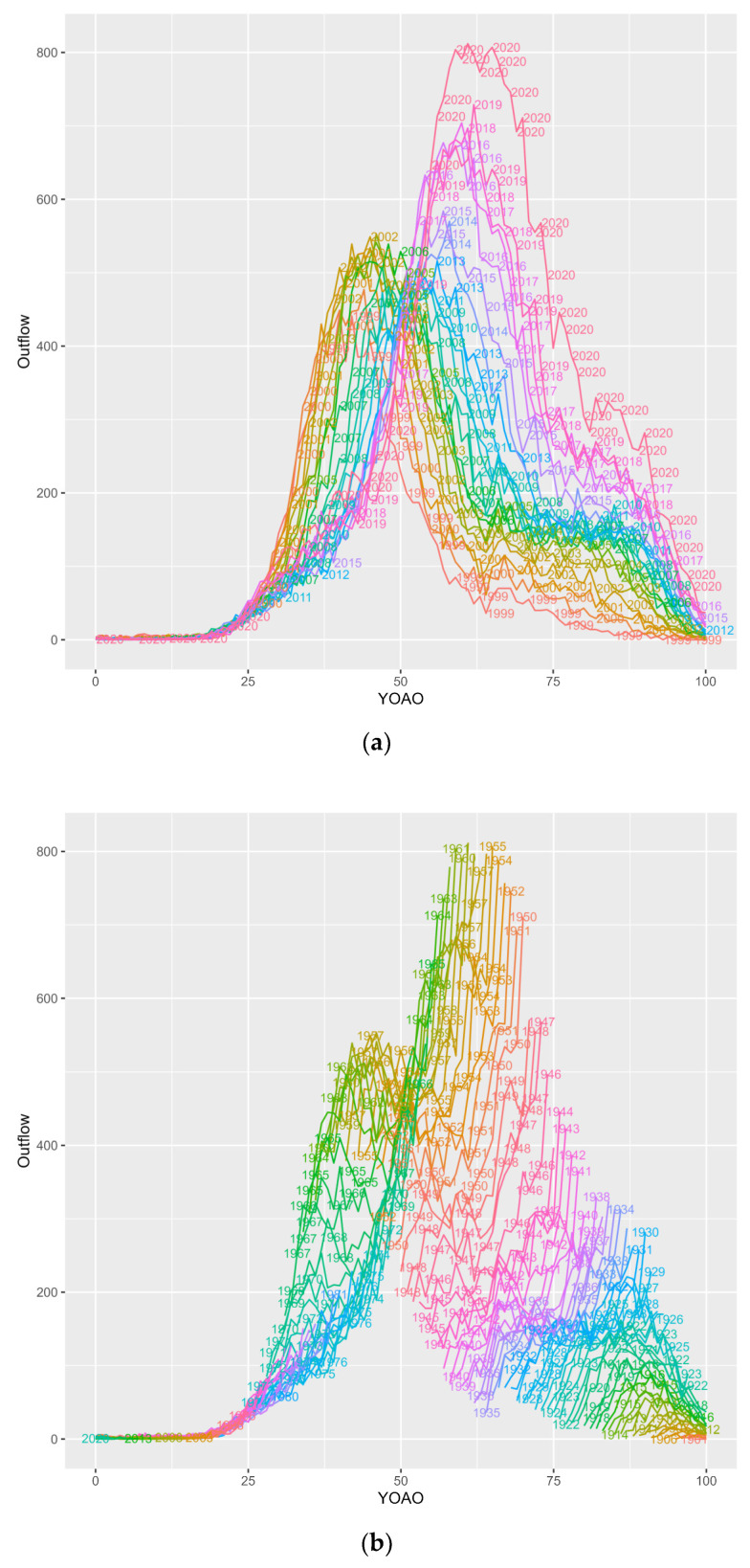

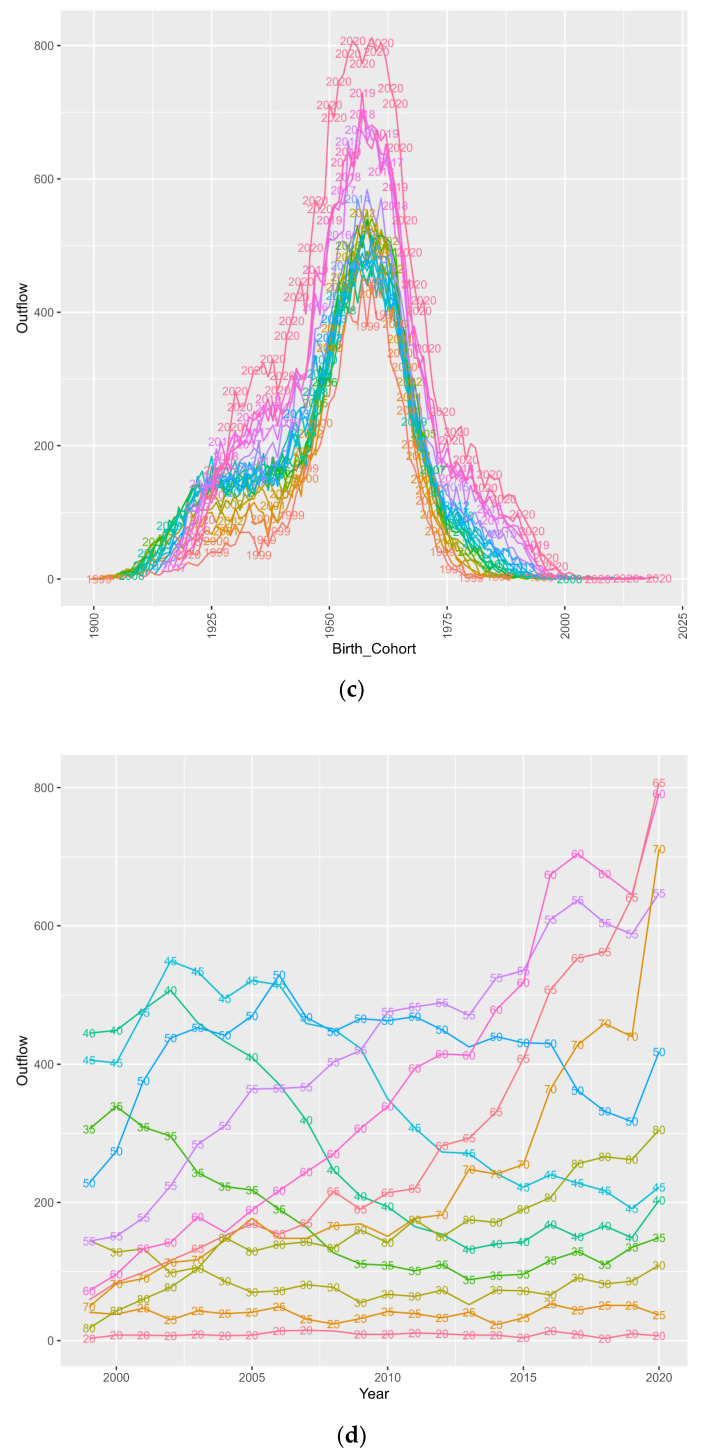

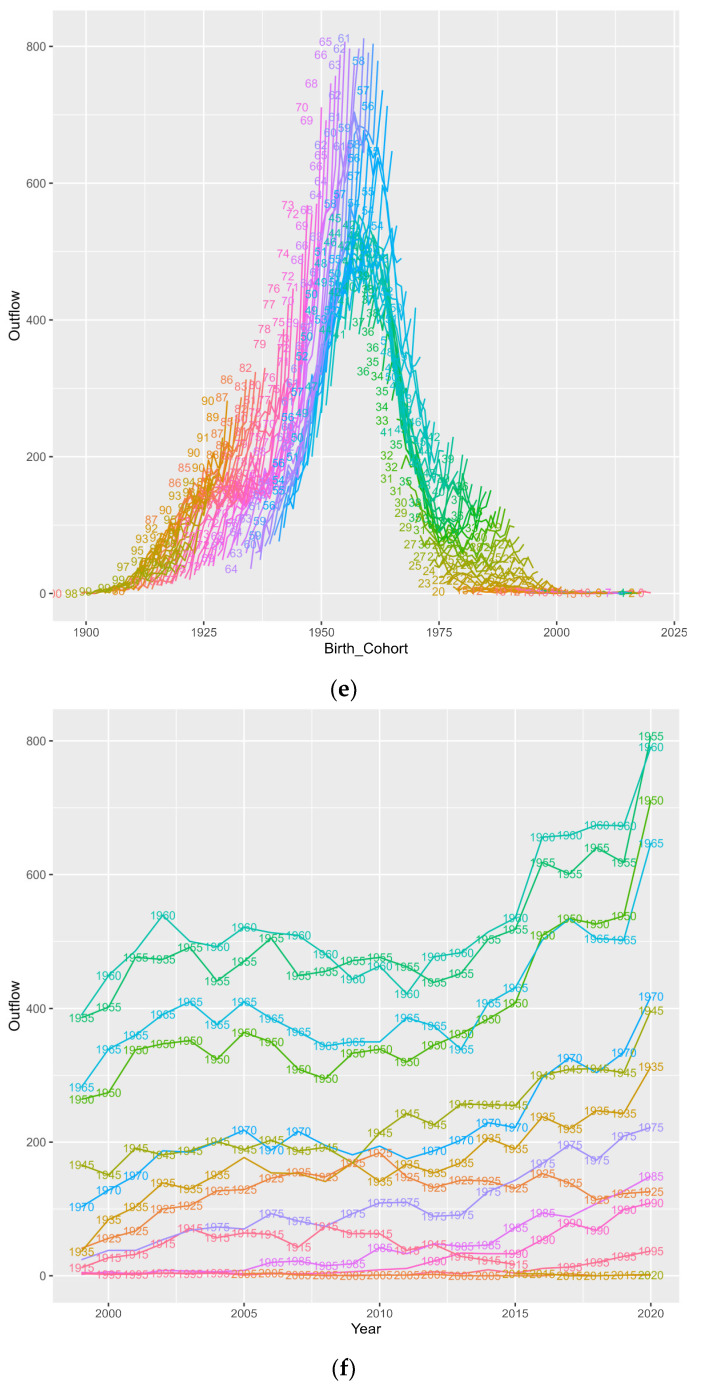
(**a**) Outflow (Deaths) among CMS HIV+ cases, YOAO in period. (**b**) Outflow (Deaths) among CMS HIV+ cases, YOAO in cohort. (**c**) Outflow (Deaths) among CMS HIV+ cases, cohort in period. (**d**) Outflow (Deaths) among CMS HIV+ cases, period in YOAO. (**e**) Outflow (Deaths) among CMS HIV+ cases, cohort in YOAO. (**f**) Outflow (Deaths) among CMS HIV+ cases, period in cohort.

Figure 6 subplots outflow, or HIV+ CMS deaths observed in this study by two factor APC plots, considering two age, period or cohort features at a time for a total of six subplots. Key points are summarized below on a subplot-by-subplot basis. Subplot 6a shows CMS HIV+ deaths by age in study period. In early study years, peaks for death are seen at 45–50 years old. In later study years, death peaks are at 60–65 years old. Note the increased mortality in 2020 starting at age 50. In later study years, decedent cases are still observed at prior study year volumes for prior peak ages 45–50. This means individuals are still dying at 45–50 in later years, but there is additional, later in life mortality observed as the study progresses.

Subplot 6b shows CMS HIV+ deaths by age in the birth cohort. Birth cohorts over the age of 50 continue to accelerate, and peak deflation is only observed after age 75. This means it is harder to survive to later ages for older birth cohorts. Younger birth cohorts who are not yet observed at YOAO 50 do not show increasing peaks (yet). This could mean that younger cases have mortality seasons (at 45–50 and at 64 years old) they have yet to experience.

Subplot 6c shows CMS HIV+ deaths by birth cohort in study period. Deaths appear uniform across birth cohorts with increasing mortality across study periods. Cases involving individuals born in 1960 are currently the center of CMS HIV mortality; however, mortality is observed at all ages.

Subplot 6d shows select CMS HIV+ deaths by study period in ages. Deaths at age 30–45 are decreasing across the study period. Deaths at 55–80 are increasing. This may reflect mortality eligibility, or case-by-case side dynamics such as underlying vulnerability or adaptation.

Subplot 6e shows CMS HIV+ deaths by birth cohort in ages. Death is birth-banded, with younger birth cohorts dying at younger ages and older birth cohorts at older ages. For example, peak death volume by birth cohort for cases of individuals born in 1960 show deaths at age 60, while younger birth cohorts have younger deaths. Very young birth cohorts have few deaths. Mortality appears ‘ongoing’ as bands do not show decreases except for some young birth cohorts who are likely to encounter more mortality seasons later in life.

Subplot 6f shows select CMS HIV+ deaths by study period in birth cohort. All birth cohorts have increasing mortality except for birth cohorts originated in the study period and cases born in 1915, who are perhaps reaching their survival maximum over the study period.

#### 3.3.4. Cases Observed in the Study

**Figure 7:**
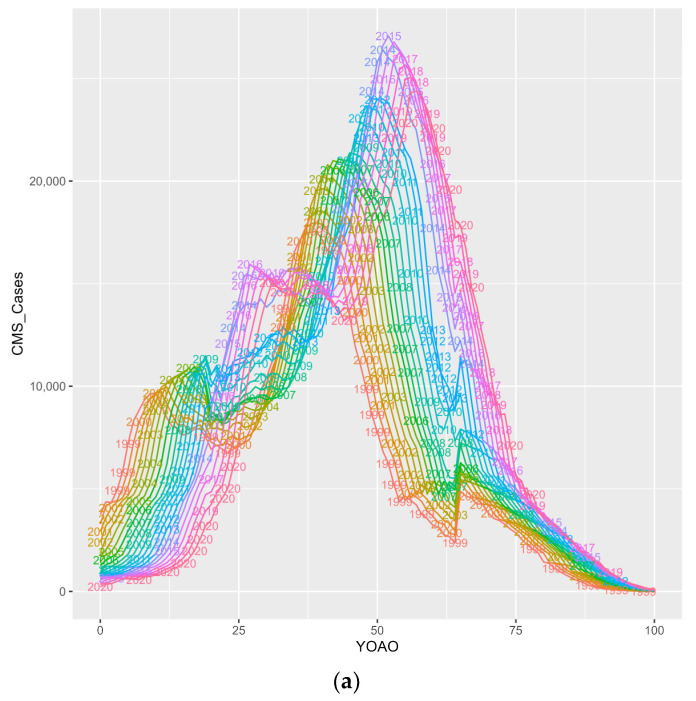

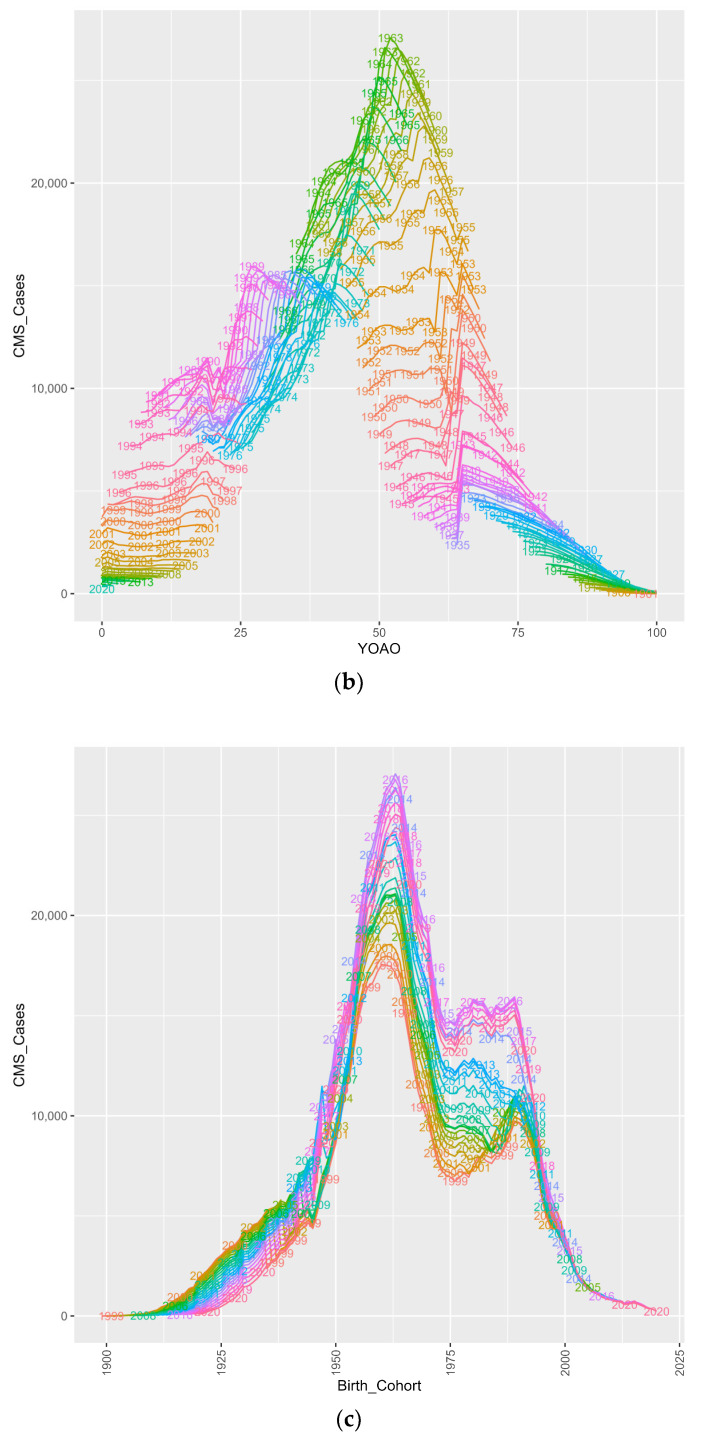

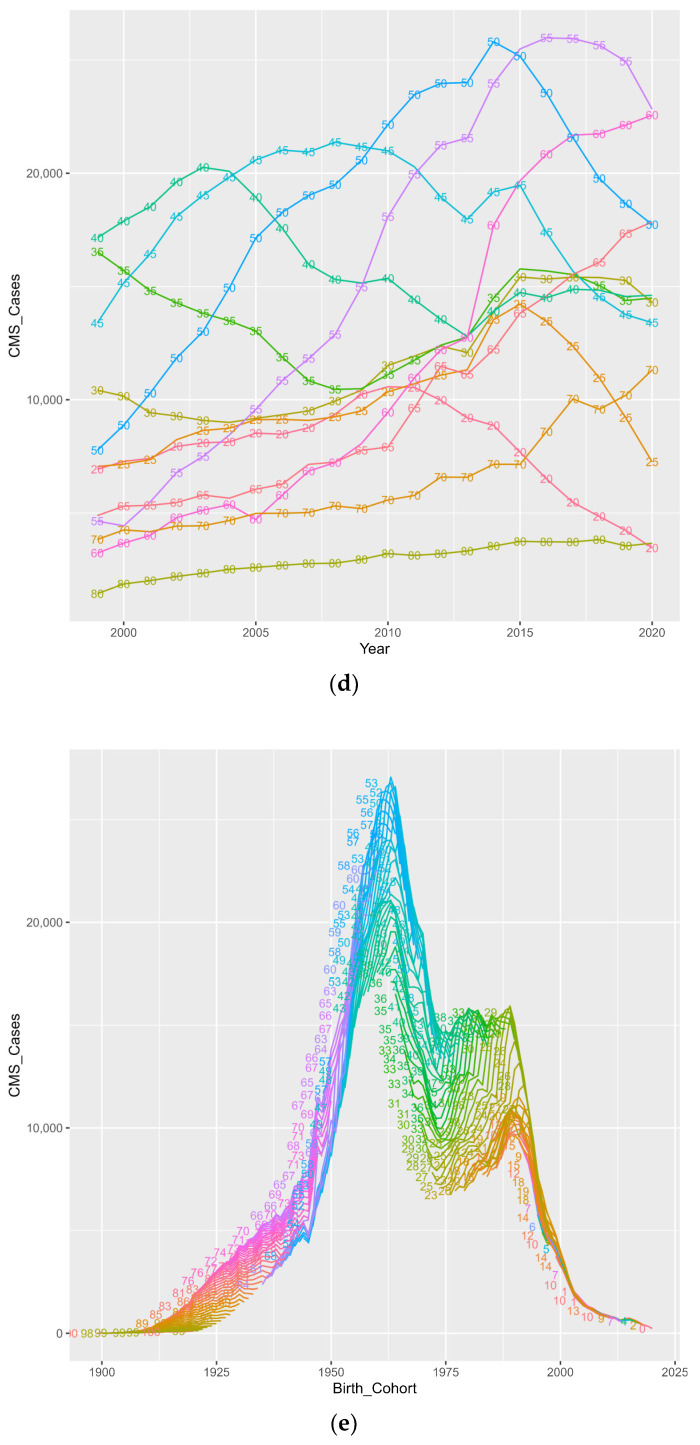

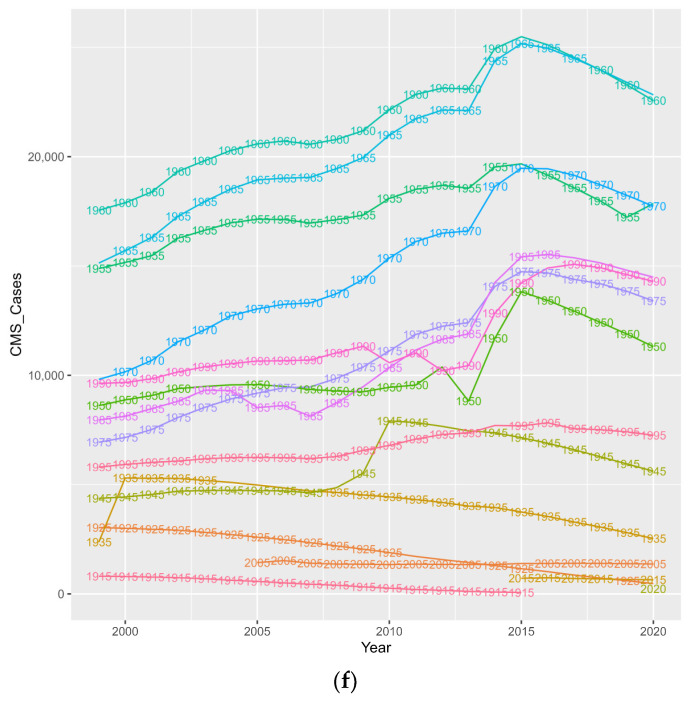
(**a**) CMS HIV+ cases, YOAO in period. (**b**) CMS HIV+ cases, YOAO in cohort. (**c**) CMS HIV+ cases, cohort in period. (**d**) CMS HIV+ cases, period in YOAO. (**e**) CMS HIV+ cases, cohort in YOAO. (**f**) CMS HIV+ cases, period in cohort.

Figure 7 subplots HIV+ CMS observed in this study by two factor APC plots, considering two age, period or cohort features at a time for a total of six subplots. Key points are summarized below on a subplot-by-subplot basis. 

Subplot 7a shows CMS HIV+ cases by age in the study period. Case observation volumes are normal, except for 2014 onward for 20- to 30-year-olds who have study year-specific volume increases. This is perhaps due to Medicaid expansion in 2014. All study years have another volume increase at age 65. This is perhaps due to Medicare enrollment at 65+. Cases present as older over time, which does not imply a survival time increase.

Subplot 7b shows CMS HIV+ cases by age in birth cohort. HIV cases have specific observational seasonality. Cases observed for those under 25, 20–45, 30–55, 50–75 and cases observed for those 65–100. These wave groups could be transmission wave groups for HIV, impacting birth cohorts at different, yet clustered ages.

Subplot 7c shows CMS HIV+ cases by birth cohort in study period. Case observation is bimodal. Millennial HIV birth cohorts have volumes that are increasingly approaching parity with the volume peak in the 1960 birth cohort.

Subplot 7d shows select CMS HIV+ cases by study period in ages. Case observation dips for 35- and 40-year-olds but recovers over the study period. For 25- and 20-year-olds, it dips and does not recover as it does for 45-year-olds. This could be partly due to decreases in pediatric infections for early ages and increases in age at infection. For 50–80-year-olds, there are increasing case volumes.

Subplot 7e shows CMS HIV+ cases by birth cohort relative to age. Peak case observation by birth cohort is for individuals born in the 1960s. They are typically observed in their 50s. Case observation volumes appear generational, with the emerging generation showing an emerging volume (1975–2000) that older generations are not observed at. Individuals born after the ‘epidemic’ started (1980s) have a wider observed age span than other birth cohorts.

Subplot 7f shows select CMS HIV+ cases by study period in birth cohorts. Cases involving individuals born after 1935 have increasing volumes generally. Pediatric cohorts are depressed (2005, 2020).

### 3.4. Poisson Linear Models

**Figure 8:**
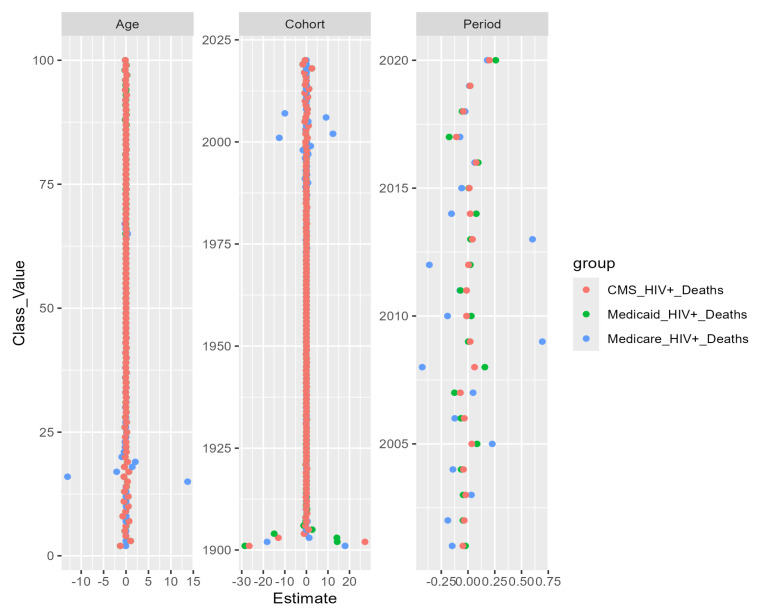
Age, period and cohort estimates from Poisson linear models for Medicare, Medicaid and CMS HIV+ decedent cases.

Two by two plots detect some variation in mortality/outflow. To locate emergent mortality stability, HIV+ decedent cases were fitted, by program, to Poisson linear models, and the effect within age, period and cohort was plotted to highlight detectable differences (Figure 8) within ages, periods and cohorts, if any. All plots and study dataset analyses were conducted in R. Values for Figure 8 were computed using the APC package [25]. A score of zero means no difference from expected prior observations, while positive and negative numerical scores highlight when changes are observed. The age panel shows variation before age 25 for Medicare, suggesting that pediatric groups may have survival differences. Younger and older birth cohorts, before 1925 for CMS and after 2000 for Medicare, also show mortality variation/instability. The time period shows Medicare-specific differences, which perhaps reflect influenza mortality.

## 4. Discussion

While treatment changes (Highly Active Anti-Retroviral Therapy) may be at the top of one’s mind in segmenting outcome eras, birth cohort effects may be more important. This is perhaps due to under-observed local effects such as AIDS deaths peaking in the US in 1995–1996, making study cases eligible for other causes of death (potentially at similar ages) while improvements in medications bring their own opportunities for AIDS avoidance as well as challenges of cost, access to care, toxicity and drug interactions [26,27,28,29,30,31,32]. Testing, mortality and disease course assessments (viral load testing) lent HIV virus-specific changes to understanding survival and case discovery [33,34,35]. These complex changes highlight the importance of taking an APC perspective when studying complexity in HIV-TRN in the United States.

The linear model did not show meaningful survival time instability between periods, ages, or cohorts, except for extremely young and extremely old cases. If birth cohort does not produce meaningful APC disambiguation, we could be witnessing an HIV endemic flowing across generations, rather than an epidemic. Decreases observed in YOAO mortality units are likely reflective of underlying case dynamics involving pediatric (collapse) or older adult (expansion) infections, rather than improvements in treatment amidst observations of decedent cases at younger and older ages. Observing pediatric cases became more difficult over the study period. This is likely due to improvements in controlling mother-to-child transmission [36,37,38,39]. The expansion of older adult cases is likely due to older adults increasingly encountering HIV risks rather than adults surviving to older ages.

Concluding that HIV is a solved problem in the United States forgoes generational HIV outcomes. This study shows millennial HIV cases and mortality burdens as well as HIV mortality burdens for adults remaining stable with endemic expansion for older adults. Given these findings, HIV may still hold some surprises for US epidemiologists, as HIV+ millennials age into adult HIV+ mortality seasons observed at middle age among older birth cohorts. As the oldest millennials are not yet 45 in this dataset, the beginning of the first adult HIV mortality season, it could be that decreases in HIV mortality observed by some projects are APC-confounded. Withstanding the progress on eradicating pediatric HIV in the United States, there are few comparable birth cohorts across treatment eras, let alone mortality waves to draw a defensible conclusion about survival time at large.

Given the consistency of birth cohort case and mortality features observed in this study, it may be better to say the US has an ‘endemic HIV’ rather than ‘epidemic HIV’ problem. The utility of using epidemic tools in leu of endemic eradication tools may be grounds for future study. The future will tell if millennial cases and mortality peak sooner than previous generations, but given the presence of decedent cases among ‘young’ people in the late study period, it is unlikely that additional aging will benefit survival prospects which are already lacking. Though not well understood, COVID-19 likely impacted mortality in 2020 HIV studies [40,41,42,43,44,45]. Future (post 2020) COVID-19 mortality may also contribute to HIV+ mortality dynamics. Aging itself has inherent risks which should not be conflated with those of younger cohorts [46].

Time periods are complex features which are often flattened or evacuated of complexity when evaluating HIV-TRN outcomes. Flattening includes ignoring eras prior to the start of the study and their implications for study events. Increases in chronic disease and the opioid crisis should have ‘all cause’ mortality effects in an HIV+ cohort [47,48]. Despite these profound non-HIV era changes, HIV survival is traditionally evaluated using a point-in-time life table model, where year of infection is assumed to be deterministic and observed or simulated mortality assumed accurate [9,49,50,51]. Such estimation approaches likely run into ceiling and floor effects, where cases achieve high or low survival years relative to an assumed infinity or HIV-matched survival time rather than observing decedent cases within birth cohorts [52].

APC disaggregation allows for the identification of model effects which can confound or mislead even skilled scientists. Attempts to evaluate outcomes, comorbidity and case volumes should disaggregate by age, birth cohort and, if known, age at infection and year of infection to avoid APC-confounding. HIV is a ‘new’ disease and there has not been a full generational cycle of hosts since the disease emerged, became epidemic and potentially endemic. Consequently, finding a ‘prior base line’ to compare outcomes without APC-confounding may prove challenging, though the use of birth cohorts may provide some assistance.

## 5. Conclusions

On some level you only die once; and, until you are dead, we do not really know if you lived longer than a similar person in a different era, let alone why you did or did not. Prior observation may inform survival expectations; however, this review of 1.5 million HIV+ cases from the United States finds varying mortality over the study period but increasing mortality within birth cohorts. Claims that HIV+ cases are ‘living longer’ without context is most likely the product of conflating the complex interplay of case age, study period, and birth cohort. Studies that do not control for multiple mortality seasons within their HIV cohorts most likely confuse signal complexity for clinical outcomes. APC may be a useful foil when considering TRN in human observation records. There are also anthropological factors worth considering when making mathematical models of or attempting to simulate human observation record systems.

## Figures and Tables

**Table 1:** HIV+ distinct individual-case attribution by diagnostic code utilization.

Study Year	Medicaid 042	Medicaid B20	Medicaid Z21	Medicare 042	Medicare B20	Medicare Z21
1999	142,664	-	-	56,090	-	-
2000	150,651	-	-	61,622	-	-
2001	159,848	-	-	66,041	-	-
2002	166,384	-	-	72,565	-	-
2003	174,245	-	-	79,110	-	-
2004	178,291	-	-	77,319	-	-
2005	180,045	-	-	75,755	-	-
2006	179,379	-	-	76,870	-	-
2007	177,276	-	-	77,966	-	-
2008	181,134	0	-	80,344	-	-
2009	191,199	-	-	83,599	-	-
2010	197,567	-	-	86,708	-	-
2011	216,568	-	-	90,071	-	-
2012	225,621	-	-	91,735	-	-
2013	259,146	-	-	92,694	-	-
2014	357,026	-	-	94,175	-	-
2015	363,461	199,374	56,738	85,309	53,901	27,541
2016	10,417	354,413	142,011	-	95,132	65,355
2017	10,323	282,717	135,929	-	95,969	62,276
2018	14	288,042	109,706	-	97,607	54,347
2019	11	293,815	123,430	-	94,155	56,237
2020	0	274,540	121,049	-	68,294	38,315

**Table 2:** Distinct cases, deaths and observation years.

1999 Through 2020	Medicare	Medicaid	Medicare and Medicaid (CMS)
HIV− Life Courses	109,925,470	207,544,824	283,688,152
HIV+ Life Courses	580,702	1,446,290	1,543,041
HIV+ Rate	0.53%	0.70%	0.54%
HIV− Deaths	45,547,376	13,596,200	48,598,568
HIV− Mortality Rate	41.43%	6.55%	17.13%
HIV+ Deaths	233,055	246,083	363,425
HIV+ Mortality Rate	40.13%	17.01%	23.55%
HIV− Obs Years	1,120,737,536	1,468,669,711	2,372,643,661
Obs Year Per HIV−	10.2	7.08	8.36
HIV+ Obs Years	6,269,195	15,748,212	17,940,305
Obs Year Per HIV+	10.8	10.89	11.63
HIV− Decedent Obs Years	457,763,732	93,979,728	480,935,555
Obs Year Per HIV− Decedent	10.05	6.91	9.9
HIV+ Decedent Obs Years	2,165,840	2,155,055	3,364,932
Obs Year Per HIV+ Decedent	9.29	8.76	9.26

**Table 3:** HIV+ life courses observed with deaths for the CMS group.

Study Year	HIV+ CMS Cases	HIV+ CMS Deaths	Mortality Rate
1999	633,663	9555	1.51%
2000	653,120	11,286	1.73%
2001	674,510	12,689	1.88%
2002	709,227	13,752	1.94%
2003	731,761	14,566	1.99%
2004	746,915	14,773	1.98%
2005	755,021	15,508	2.05%
2006	757,359	15,717	2.08%
2007	753,680	14,822	1.97%
2008	766,146	14,856	1.94%
2009	786,178	15,192	1.93%
2010	824,100	15,321	1.86%
2011	850,870	15,230	1.79%
2012	864,101	15,213	1.76%
2013	858,419	15,838	1.85%
2014	948,523	16,833	1.77%
2015	981,513	18,104	1.84%
2016	973,816	21,009	2.16%
2017	955,187	21,874	2.29%
2018	932,512	21,779	2.34%
2019	905,949	22,110	2.44%
2020	877,735	27,398	3.12%

## Data Availability

The study dataset is available upon request to the corresponding author at nick.williams@nih.gov. For identifiable or encounter-level CMS data, requests should be made through https://www2.ccwdata.org/web/guest/home/ (accessed on 3 November 2024).
